# Facilitative effect of awe on cooperation: The role of the small‐self and self‐other inclusion

**DOI:** 10.1002/pchj.730

**Published:** 2024-02-01

**Authors:** Qin Wu, Liying Cui, Xianguo Han, Yanxia Wu, Wen He

**Affiliations:** ^1^ College of Psychology Shanghai Normal University Shanghai China; ^2^ Counseling and Student Development Center Shanghai Normal University Shanghai China

**Keywords:** awe, cooperation, self‐other inclusion, self‐transcendence, small‐self

## Abstract

Awe has been theorized as a kind of self‐transcendence emotion that has an important impact on individual social behavior. Based on the self‐transcendence of awe, this study examined how awe can increase small‐self and self‐other inclusion to facilitate cooperation among individuals across three studies (*N* = 1162). First, the relationship between awe, cooperative propensity, and the mediating role of small‐self and self‐other inclusion in the relationship was examined using questionnaires on trait levels (Study 1). Second, awe emotions were induced from the state level through behavioral experiments to verify the facilitative effect on cooperative behavior in multiple rounds of public goods dilemma (Study 2). Third, by adding the induction of negative awe to discuss the impact of different valence of awe on cooperative behavior, the mediating role of small‐self and self‐other inclusion was supported (Study 3). Results show that awe has a facilitative effect on cooperation, which provides strong evidence for the positive social function of self‐transcendent emotional awe.

## INTRODUCTION

Awe refers to the emotional experience of wonder in the face of something vaster, greater, and beyond current comprehension (Keltner & Haidt, 2003). As an emotion of self‐transcendence, awe has a broad yet positive impact on individual mental health and social behavior (Pizarro et al., 2021), and the social function of awe has triggered continuous exploration by psychologists in recent years.

Cooperation is a fundamental topic in the evolution and development of human society (Sjåstad, 2019), serving as a vital form of social interaction during the process of adolescent socialization. Cooperation refers to relatively stable, persistent, and implicit personality dispositions (i.e., cooperative propensity) as well as immediate, impulsive, and outwards behavioral decisions (i.e., cooperative behavior; Liu, 2021). Previous studies have characterized the relationship between awe and prosocial behaviors such as helping, generosity, and altruism (Lin et al., 2021; Piff et al., 2015), or by pro‐environmental behaviors (Zhao et al., 2018). However, cooperation differs from prosocial behavior in terms of behavioral motivation, interactive features, and beneficial results, which is usually motivated by reciprocity or a win‐win situation, and individuals maximize the benefits of their behavior by compromising on short‐term self‐interest in interaction. Furthermore, previous studies have paid more attention to or tacitly discussed positive awe, and explored and confirmed the facilitative effect of awe on social behavior. While extensive research describes valence as the central element of an emotion that can guide behaviors, recent years of research have indicated that negative awe experiences are relatively less positive and have a distinct emotion appraisal profile (Chaudhury et al., 2021; Gordon et al., 2017). However, the role of negative and positive valence in the influence of awe on cooperation and whether the influence paths are consistent is far from clear.

Analyzing the self‐transcendence of awe, self‐transcendence includes two broad complementary subcomponents: (a) an “annhilational” component, which refers to the reduced self‐salience; and (b) a “relational” component, which refers to the sense of connectedness, even to the point of oneness, with something beyond the self (Yaden et al., 2017). The “annhilational” component may reduce the negative aspects of excessive self‐focus while the “relational” component likely involves processes related to perceived social connection (Yaden et al., 2017). Based on this, we propose a path model of how awe affects cooperation, and combine questionnaire research and behavioral experiments to comprehensively examine the empirical relationship between awe and cooperation by exploring the reduced self‐salience and increased sense of connectedness mediators. Specifically, we investigate the role of small‐self and self‐other inclusion, and explore whether there is a difference in the effect of negative (positive) awe valence on cooperation in this paper.

### Awe as a facilitator of cooperation

When something is vast beyond an individual's ordinary frame of reference and requires adjusting one's mental structure to assimilate what is being perceived, this results in a complex and powerful emotional response of awe, characterized by the perceived vastness and the need for accommodation (Keltner & Haidt, 2003). The experience of awe places the self in secondary position, shows a higher concern for the welfare of others, and has a wide range of positive effects on individual social behavior (Piff et al., 2015; Prade & Saroglou, 2016; Stellar et al., 2018).

Prior research highlights emotion's crucial role in cooperation. Moral emotions such as shame (Sznycer et al., 2016), guilt (Kahn et al., 2016), and embarrassment (Müller‐Pinzler et al., 2015) can promote wide‐ranging cooperative behavior. Further, positive emotions are also conducive to cooperative interaction. Trait gratitude and experiment‐induced gratitude can significantly improve the level of individual cooperation (Ren et al., 2021). Empathy has also been recognized as an effective tool to deal with misunderstanding behaviors, thereby maintaining or strengthening cooperation (Rumble et al., 2010).

As a complex emotional experience, theoretical and empirical accounts of awe categorized it as a moral and positive emotion (Piff et al., 2015; Stellar et al., 2017), one that is both prosocial and self‐transcendent (Pizarro et al., 2021; Rankin et al., 2020). Consequently, similar to other moral and positive emotions, awe provides a theoretically reliable prediction for cooperation. Grounded in self‐transcendent attributes and functions, self‐transcendent emotion simultaneously orients the individual toward the welfare of others and motivates collective action for the greater good (Pizarro et al., 2021; Stellar, 2021). Additionally, it empowers individuals to be less concerned about immediate benefits and costs, enabling them to resist the temptation of temporary interests (Wang et al., 2019) – all of which play pivotal roles in facilitating cooperation.

Furthermore, perceptual vastness is one of the core characteristics of awe. A sense of awe makes people feel as if they are part of something larger than themselves while simultaneously placing less significance on their own self‐interests, and enhancing the individual's goodwill towards the external world, the community, and society at large (Bai et al., 2017; Stellar et al., 2017; van Cappellen et al., 2013). Another core characteristic is the need for accommodation, which represents the process of mental reconstruction when the new experience does not align with the existing mental structure. Researchers have underscored that the key to solving the problem of cooperation is to encourage collective interests to take precedence over personal interests and find a balance in the relationship (Stellar et al., 2018). The mental reconstruction process induced by experiencing awe allows individuals to change their perspectives and view the relationship between the individual and the collective interests from a larger and longer‐term perspective—instead of merely focusing on short‐term self‐interests—which is conducive to promoting mutual cooperation (Li et al., 2019; Stellar et al., 2017).

Therefore, based on the emotional categorization of awe, the attributes of self‐transcendence and its core characteristics, this study proposes the following hypothesis:

Hypothesis 1 Awe can facilitate cooperation in social interactions.

### Role of the small‐self

Awe is other‐oriented rather than self‐oriented, and extols the benefits of letting the ego recede into the background. No matter how awe is defined and classified, it is obvious that awe has a remarkable impact on the self. People have reported consistently that awe makes them perceive themselves as small or even insignificant, allowing for the emergence of a sense of self‐diminishment (Bai et al., 2017; Chen & Mongrain, 2020; Stellar, 2021), thus changing the perspective of self (from the perspective of a “unique self” to a “general self,” and from the perspective of an “individual self” to a “collective self”) (Rankin et al., 2020; Shiota et al., 2007).

When people shift their attention away from themselves and change the perspective of self, they behave more inclusively and generously by paying attention to and aligning with the feelings and needs of others (Stellar, 2021), and their collective orientation will increase accordingly (Horberg et al., 2011). Moreover, many studies also demonstrate that a sense of the small self serves as an important mechanism, underlying the prosocial effects of awe in various social contexts (e.g., Keltner & Haidt, 2003; Piff et al., 2015; Prade & Saroglou, 2016).

Therefore, given that awe can trigger a relative diminishment of the individual self that favors cooperative interactions by increasing focus on others or collective interests, this study proposes the following hypothesis:

Hypothesis 2 The small‐self mediates the relationship between awe and cooperation.

### Role of the self‐other inclusion

Self‐other inclusion refers to the individual's acceptance and understanding of others' concepts and resources to varying degrees, resulting in the overlapping between information represented on one's self and others (Aron et al., 1991), which emphasizes the relational self that has similar representations of self and others, and describes states of interpersonal connection (Aron et al., 2004).

Awe challenges individuals to look beyond themselves, offering new insights and a wider perspective filled with a deeper sense of connection, meaning, and purpose (Stellar, 2021). Moreover, it is believed that awe has the power to drive people to combine individual and broad social entities such as the nations or the human communities that affect all of humanity (Keltner & Haidt, 2003; Stellar, 2021). As a self‐transcendent emotion, these downstream consequences of awe experiences produce a greater sense of connection with what surrounds us and prompt individuals to form a collective and inclusive identity (Pizarro et al., 2021), which in turn fosters the integration with others and the external world. Other empirical research also showed that the awe induced by watching videos of magnificent natural landscapes caused participants to feel more connected to people in general on the Inclusion of the Other in the Self Scale (van Cappellen & Saroglou, 2012).

Based on social identity theory and the results of meta‐analysis in the field of cooperation, inclusive or common identity is an important facilitator of cooperation among individuals (Balliet et al., 2014; Tajfel, 1974). The increased levels of self‐other inclusion between an individual and others increases the intimate perception of their relationship and strengthens the “sense of us and community” that can be formed and the consideration of the interests of others as equally important as their individual interests (Zi & He, 2019), thereby facilitating cooperative and reciprocal behavior.

Therefore, this study proposes the following hypothesis:

Hypothesis 3 The self‐other inclusion mediates the relationship between awe and cooperation.

### Awe of different valence

Awe can be both wonderful and terrifying, encompassing both “reverence” and “fear”. When individuals confront immense and threatening stimuli, such as destructive natural disasters or punitive divine forces, they experience negative awe accompanied by a sense of fear (Shiota et al., 2007). Existing work in recent years demonstrated that emotional experiences of positive and negative valence awe are different and might have different downstream outcomes (Chaudhury et al., 2021; Gordon et al., 2017). Specifically, positive awe typically involves certain positive emotional aspects, such as surprise, curiosity, and wonder, while negative awe was more strongly associated with feelings of fear and anxiety, reduced appraisals of personal control and greater appraisals of situational control (Gordon et al., 2017; Zhao et al., 2021).

However, numerous studies have indicated that negative awe, triggered by stimuli perceived as immense, can also promote a sense of small self, enhance concern for others, and consequently stimulate prosocial behaviors such as charitable acts and helping behaviors (Piff et al., 2015; Seo et al., 2022; Wang et al., 2022). Simultaneously, based on the model of group‐based control, when individuals experience a loss of control, they are inclined to respond by identifying with the group or in terms of “we” instead of “I”, in order to address the limitations of their personal control (Fritsche et al., 2013). Empirical studies also indicate that threat‐based awe highlights people's sense of global citizenship (Seo et al., 2022) or global community (Wang et al., 2022), enhancing their experience of a stronger sense of connection.

In the context of a profound sense of insignificance, individuals shift their focus from self‐oriented to concern for the collective aspects of self that are integrated into social communities (Seo et al., 2022). In the context of a highly inclusive human identification, individuals no longer act solely as individuals but rather as members of a collective, thereby safeguarding collective interests (Fritsche & Masson, 2021). Based on this, we propose the following hypotheses:

Hypothesis 4 Both negative and positive awe can promote cooperation among individuals.

Hypothesis 5 This effect is indirectly mediated through small‐self and self‐other inclusion.

### Current research

As a self‐transcendent emotion, awe can weaken an individual's attention to self‐interest and increase the propensity for thinking about and taking into account the needs of others or the collective (Prade & Saroglou, 2016). However, individuals who are more prone or predisposed to experiencing awe often experience awe more frequently (Quesnel & Riecke, 2018). While awe as an emotional state encompasses both positive and negative valence, previous research has predominantly focused on positive awe. Therefore, the present studies comprehensively explore the influence of awe on cooperation from various dimensions, including emotional traits and states as well as positive and negative valence. Furthermore, building upon the two subcomponents of awe's self‐transcendence attributes, we propose that awe influences cooperation through the mediators of reduced self‐salience and increased sense of connectedness, and employ a combination of questionnaire and behavioral experiments to test this mediation model.

Further, based on the analysis of the influence of awe on social behavior and its self‐transcendence, it is assumed that people have higher traits of awe (Study 1) and those who experienced either positive or negative awe induction (Study 2 and Study 3) have a higher level of cooperation to comprehensively verify H1 and H4. Moreover, we examine H2, H3, and H5 by assuming that small‐self and self‐other inclusion mediate the relationship between awe (traits and states, positive and negative valence) and cooperation (Study 1 and Study 3).

## STUDY 1

Study 1 uses a questionnaire measurement method to explore the relationship between awe and cooperation propensity, and the role of small‐self and self‐other inclusion in their relationship from the context of trait levels.

### Method

A total of 982 students from a high school in Zhejiang, China, participated in the online questionnaire survey. The questionnaires link was sent to class groups online, including the parents of the students by the head teacher, and the students used their mobile phones to fill out the questionnaire in the classroom. Questionnaires that had the same answer consistently or where all three test questions (specify the answer to the question such as “*Please choose 1 for this question*”) were wrong, deemed invalid and excluded; ultimately, 907 questionnaires remained (506 males, age: *M* = 16.08, *SD* = 0.76, range: 15–19 years). The effective response rate of the questionnaire was 92.4%, which included 478 10th grade students (52.7%) and 429 11th grade students (47.3%).

The data were collected using an anonymous form through a web link on the platform “QUESTIONSTAR”. Upon reading the instructions and providing their signed informed consent forms, the participants completed the questionnaires within 15 min. The participants could also choose to not participate in the survey and submit the questionnaire directly; they also had the right to withdraw at any time during the survey. All data were collected after approval from the Ethics Review Board of the Social Sciences Office of Shanghai Normal University.

#### Measures

##### Awe

The Dispositional Awe Scale was compiled by Shiota et al. (2006) to measure awe, which is a sub‐scale of the Trait Positive Emotion Scale containing six items (e.g., “*I often have a feeling of awe*”). The Chinese version of the scale has demonstrated satisfactory construct validity and internal reliability (Zhao et al., 2018). In the current study, all items (*α* = .87) were scored on a seven‐point Likert scale, ranging from 1 (*absolutely disagree*) to 7 (*absolutely agree*). The higher the total score of the items, the higher the level of an individual's dispositional awe.

##### Cooperative propensity

Cooperation was assessed using the 13‐item cooperation personality subscale compiled by Xie et al. (2006), which covers content across three dimensions, namely, inclusiveness, reciprocity, and gregariousness. The items (e.g., “*I believe that cooperation is more helpful in improving performance than the competition*”) were rated on a 5‐point Likert scale, ranging from 1 (*totally disagree*) to 5 (*totally agree*). This measure has previously been proven reliable and valid in a Chinese sample (Ren et al., 2021; Xie et al., 2006). In this study, the α coefficient of the scale was .90, and the *α* coefficients of inclusiveness, reciprocity, and gregariousness were .87, .76, and .81, respectively.

##### Small‐self

Using the simplified version of the small‐self questionnaire to measure the level of small‐self, participants rated their agreement with four statements (e.g., “*I feel small or insignificant*”) from 1 (*absolutely disagree*) to 7 (*absolutely agree*) (Huta & Ryan, 2010; Shiota et al., 2007). These four items (*α* = .68) tapped perceptions of vastness vis‐à‐vis the self and the accompanying sense of smallness, and they formed a reliable measure of the small‐self (Piff et al., 2015).

##### Self‐other inclusion

The Inclusion of the Other in the Self Scale (Aron et al., 1992) was used, which is a pictorial measure of interpersonal interconnectedness and is currently the most widely used measure of self‐other inclusion (Myers et al., 2014). The scale is composed of diagrams, each representing different degrees of overlap of two circles representing the self and the other. Participants were told to choose the best diagram from a pair of circles that start with no overlap and end in almost total overlap, to describe the relationship between themselves and others (a total of 7 pairs of circle diagrams); the greater the degree of overlap between the two circles, the greater the degree of inclusion between oneself and others.

### Results and discussion

#### Common method bias effect

A single‐factor confirmatory factor analysis model approach was used to examine common method bias (Mossholder et al., 1998). The number of common factors was set as 1, and lower likelihood of common method bias was supported by lack of fit of the single‐factor CFA model (*χ* ^2^/*df* = 14.32, comparative fit index (CFI) = 0.68, Tucker‐Lewis Index (TLI) = 0.65, root mean square error of approximation (RMSEA) = 0.12), which suggests that the common method bias effect is not seriously problematic for the current study.

#### Descriptive statistics

The descriptive statistics and zero‐order correlations for the relevant variables are presented in Table 1. The results showed that trait awe was positively correlated with small‐self, self‐other inclusion, and cooperative propensity (*r* = .15–.58, *p*s < .001); and small‐self was significantly positively related to cooperative propensity (*r* = .14, *p* < .001), but not significantly related to self‐other inclusion (*r* = −.01, *p* > .05), and self‐other inclusion was significantly positively related to cooperative propensity (*r* = .48, *p* < .001). Gender was negatively correlated with self‐other inclusion and cooperative propensity (*r* = −.10– −0.13, *p*s < .01), and age was negatively correlated with small‐self, self‐other inclusion, and cooperative propensity (*r* = −.08– −.10, *p*s < .05).

**TABLE 1:** Means (*M*), standard deviations (*SD*), and zero‐order correlations for key variables (*N* = 907).

Variable	1	2	3	4	5	6	7	8	9
1. Gender (0, 1)									
2. Age (years)	.03								
3. Trait awe	−.06	−.09**							
4. Small‐self	.04	−.01	.15***	1					
5. Self‐other inclusion	−.13***	−.10**	.36***	−.01	1				
6. Cooperative propensity	−.10**	−.08*	.58***	.14***	.48***	1			
7. Inclusiveness	−.05	−.11***	.56***	.09**	.41***	.89***	1		
8. Reciprocity	−.11**	.01	.45***	.20***	.39***	.83***	.55***	1	
9. Gregariousness	−.10**	−.09**	.51***	.06	.45***	.91***	.78***	.62***	1
*M*	0.56	16.08	5.47	4.28	4.34	3.83	4.10	3.33	3.98
*SD*	0.50	0.76	1.21	1.37	1.98	0.68	0.70	0.86	0.78

*Note*: Coding of gender: 0 = female, 1 = male.

* *p* < .05;

** *p* < .01;

*** *p* < .001.

#### Multiple mediation model testing

**FIGURE 1:**
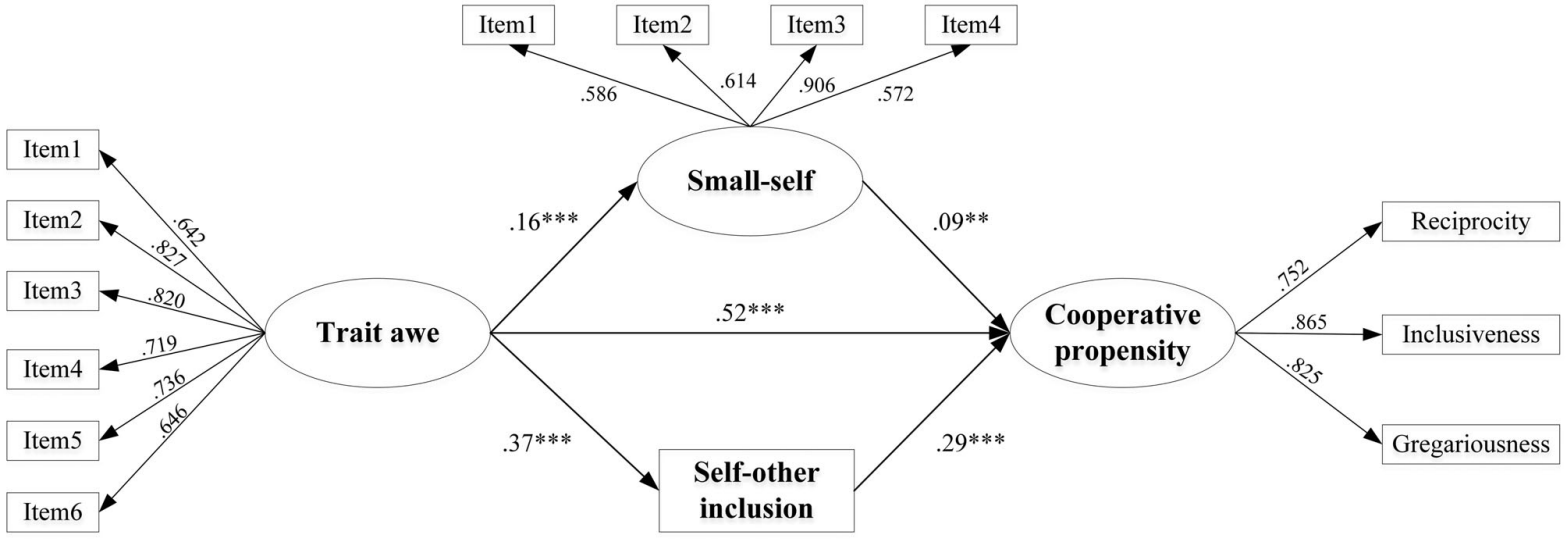
Parallel mediating effect of small‐self and self‐other inclusion between trait awe and cooperative propensity.

The Latent Structural Equation Model was adopted to examine whether small‐self and self‐other inclusion would mediate the relationship between trait awe and cooperative propensity. The model fitting test results showed that the parallel mediation model fitted the data well, *χ* ^2^/*df* = 3.99, CFI = .937, TLI = .912, RMSEA = .057. After controlling for gender (0 = female, 1 = male) and grade, the test results of the mediation model (see Figure 1) showed that trait awe significantly and positively predicted small‐self (*β* = .16, *p* < .001, 95% confidence interval [CI] = [.06, .25]) and self‐other inclusion (*β* = .37, *p* < .001, 95% CI = [.30, .43]). After adding the mediating variable of small‐self and self‐other inclusion, small‐self (*β* = .09, *p* = .006, 95% CI = [.03, .16]) and self‐other inclusion (*β* = .29, *p* < .001, 95% CI = [.22, .37]) were significant and positive predictors of an individual's cooperative propensity. The direct effect of trait awe on cooperation propensity was .52 (*p* < .001, 95% CI = [.45, .59]) and the total effect was .65 (*p* < .001, 95% CI = [.58, .71]); thus, H1 was validated. The predictive effect of trait awe on individual cooperative propensity was partly mediated by the small‐self and self‐other inclusion; thus, H2 and H3 were further supported. The decomposition pathways of the mediation effects are shown in Table 2.

**TABLE 2:** Decomposition of the effect of trait awe on cooperative propensity.

Model pathways	Standardized effect size	Effect proportion	95%CI	
			LLCI	ULCI
(dir) Trait awe → Cooperative propensity	0.524	81.1%	0.453	0.594
(ind1) Trait awe → Small‐self → Cooperative propensity	0.014	2.2%	0.001	0.028
(ind2) Trait we → Self‐other inclusion → Cooperative propensity	0.108	16.7%	0.078	0.138
Total effect (dir + ind1 + ind2)	0.646	—	0.581	0.711

Abbreviations: CI, confidence interval; dir, direct path; ind1, indirect path 1; ind2, indirect path 2; LLCI, lower level of confidence interval; ULCI, upper level of confidence interval.

These results support H1, H2, and H3 from the trait level, confirming the facilitative relationship between awe and cooperative propensity, as well as the parallel mediating role of small‐self and self‐other inclusion. However, the cooperative propensity reflects the possibility of an individual to choose cooperation in a potential cooperative situation. Individuals with a high cooperative propensity are more likely to satisfy long‐term collective interests by suppressing short‐term self‐interests and making choices in terms of actual behaviors. Nonetheless, the process of transforming inclination into behavior is also subject to many factors. Therefore, Study 2 explored the effect of awe as an emotional state on individual cooperative behavior by inducing awe in the experiment and adopting the public goods paradigm of cooperative behavior.

## STUDY 2

Study 2 aimed to generalize the results of the relationship between awe and cooperation in Study 1 using behavioral experiments to induce awe in participants through video clips, and further explore the facilitative effect of awe on individual cooperation from the emotional state level.

### Method

#### Participants and procedures

Before the experiment, a moderate (*f* = .30) effect size was adopted to calculate the sample size by G*Power 3.1 (Faul et al., 2009). The sample size analysis of variance (ANOVA) (fixed effects, omnibus, one‐way, *α* = .05, and 80% power) showed that a minimum of 90 participants were required.

In this study, 116 10th grade students were recruited from two high schools in Shanghai, China, to participate in the experiment. Excluding the 12 invalid participants who did not complete the experiment due to glitches in the experimental procedure, a total of 104 valid participants were retained (53 females, 51 males). The effective response rate for the participants was 89.7%, of which 54 were in the experimental group and 50 were in the control group.

The recruitment information was disseminated by the head teacher in the online class group. The experiment was performed in a quiet and spacious laboratory at the participants' school, which was pre‐installed with eight computers connected to headphones. The experiment was conducted with eight participants each time (four males and four females) simultaneously under the same experimental conditions. After entering the laboratory, the investigator briefly introduced the experimental purpose and procedure and emphasized the anonymity and precautions of the whole experiment (e.g., do not communicate with each other, do not click on other pages of the computer). Then, the participants filled out the informed consent forms. The participants could choose not to answer certain questions and could withdraw from the experiment at any time, and were assured that the experimental data would not be disclosed to anyone except the researchers.

Thereafter, the participants wore headphones to watch a 4‐min video in full‐screen mode on the computer to induce emotion (the awe‐conditioned group watched clips from the BBC documentary “Planet Earth” and the neutral‐conditioned group watched an origami video) (Piff et al., 2015; Zhao et al., 2018). After watching the video, participants immediately filled in the multi‐dimensional emotional scale according to their current emotions, and finally clicked the link to log in to the game lobby and were randomly assigned to a game room with three players to complete the online public goods dilemma game. Participants received a small gift for participating after the experiment was completed.

#### Experimental materials

##### Video material

For the awe condition, participants watched a 4‐min video clip of spectacular natural landscapes (e.g., mountains, waterfalls, jungles, caves) from the BBC documentary “Planet Earth,” and a 4‐min origami paper crane video for the neutral condition.

##### Current emotion states

Referring to previous studies, the multi‐dimensional emotional scale was used to measure the current emotions of the participants after emotion‐inducing operations, which included the emotions of anger, sadness, disgust, fear, happiness, and awe. The rating scale ranges from 1 (*absolutely none*) to 7 (*very strong*), with higher numbers indicating a greater intensity of the emotion (Piff et al., 2015; Prade & Saroglou, 2016).

##### Cooperative behavior

The experiment creates game web links according to the public goods dilemma paradigm to examine the cooperative behavior of the participants. After reading the rules of the game and passing three rule comprehension questions, the participants entered the formal game. The game rules are as follows: every four participants randomly form a temporary group, at the beginning of the game, each group member has 100 yuan of game coins, the group has a public account, and the participants will put a certain amount (0–100) of game coins into the public account according to their own wishes. If the total amount of the public account is >200 yuan (contains 200), it will be doubled and distributed equally to each member of the group. Otherwise (<200 yuan) the system will not return the game coins invested by all group members (Cui et al., 2017). In each round, the participants will receive a principal of 100 yuan. After the investment of the group members, the system will automatically provide feedback on the participant's benefits in the current round and the total amount in their personal account. Overall, there were 12 rounds of investment. The first round of investment and the average investment of 12 rounds in the public goods dilemma game were considered indicators of cooperative behavior (Cui et al., 2017).

### Results and discussion

#### Manipulation checks

Compared with the neutral emotion condition (*M* = 1.48, *SD* = 1.05), participants in the awe condition (*M* = 3.78, *SD* = 1.93) experienced significantly greater feelings of awe [*F*(5,102) = 25.08, *p* < .001]. Ratings of the other five emotions did not differ significantly between the two conditions, which confirmed that the experimental operation to induce awe was effective.

#### Influence of awe on cooperative behavior in the dilemma of public goods

**FIGURE 2:**
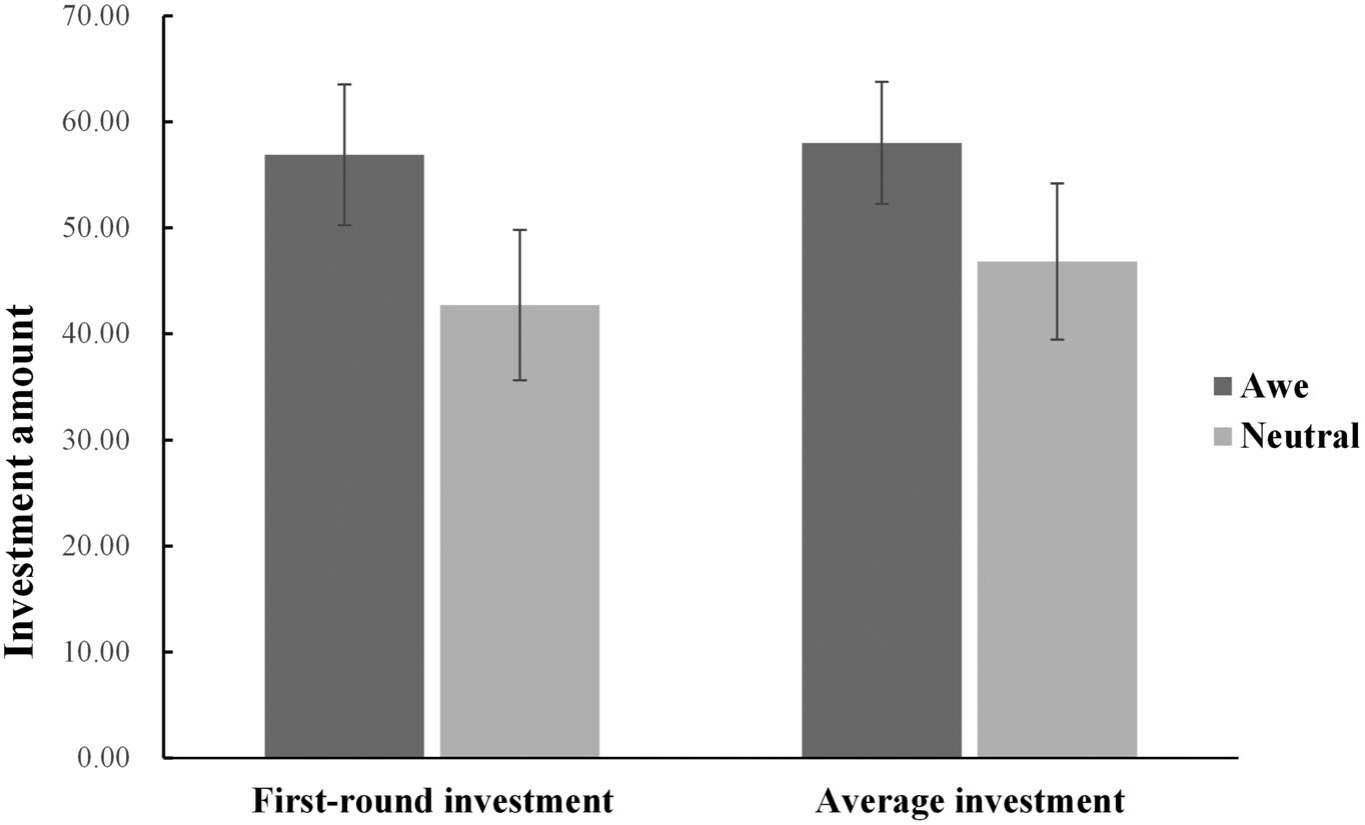
Average investment of 12 rounds and the first‐round investment of awe and neutral group. The error bar in the figure represents the standard error.

On the premise of controlling gender, a multivariable ANOVA was carried out with the group as the independent variable, with the first round of investment and the average investment of 12 rounds as dependent variables. The results showed that in the first round of investment [*F*(1,101) = 9.00, *p* = .003, *η* ^2^ = .08] and the average investment of 12 rounds [*F*(1,101) = 6.83, *p* = .010, *η* ^2^ = .06], the main effect of the group was significant, in which the investment amount in the awe group was significantly higher than that in the neutral group (*M*_first round of awe_−*M*_first round of neutral_ = 14.33; *M*_average 12‐rounds of awe_−*M*_average 12‐rounds of neutral_ = 11.59), which shows that the emotional state of awe can significantly improve the level of individual cooperation in the interaction of public goods dilemma (see Figure 2 for the investment amount distribution of different groups). The results of the sensitivity analysis (power = .80, *α* = .05) indicated that the effect size of the experimental group (*η* ^2^ = .104) was greater than the lowest detectable effect size for this sample size (*η* ^2^ = .095).

Study 2 adopted the public goods experimental paradigm of multiple rounds of real interaction and further supported the facilitating effect of awe on cooperation by inducing awe in the experimental situation. However, the baseline emotions for different groups and the impact of negative awe on cooperation were not measured in the existing research, and how awe affects cooperation has not yet been verified in the experimental situation. Therefore, Study 3 considered these limitations and used the behavioral experiment method to continue to explore the influence of different valence of awe on cooperative behavior and its psychological mechanism from the emotional state level.

## STUDY 3

Study 3 adopted the behavioral experiment method to induce positive and negative awe emotions by watching videos and explored the influence of different valence of awe on individuals' cooperative behavior in the public goods dilemma and their influence pathways.

### Method

The moderate (*f* = .30) effect size was adopted to calculate the sample size using G*Power 3.1 (Faul et al., 2009). The sample size ANOVA (fixed effects, omnibus, one‐way, *α* = .05, and 80% power, the number of groups is 3) showed that a minimum of 111 participants was required.

A total of 164 high school students were recruited from a high school in Zhejiang, China, to participate in the experiment. Excluding the 13 invalid participants who did not complete the experiment due to glitches in the experimental procedure, a total of 151 valid participants were retained (82 males, 69 females; age: *M* = 16.29, *SD* = 0.69, range: 15‐18 years). The effective response rate for the participants was 92.4%. Among them, there were 55 participants in the neutral emotion control group, 49 participants in the positive awe experimental group, and 47 participants in the negative awe experimental group.

The experiment was conducted by three psychology graduate students in a quiet, spacious laboratory at the participants' school, where 16 computers were installed in advance and connected to headphones. A total of 12 participants (the number of male and female students was as balanced as possible) were tested at the same time. Participants provided informed consent forms and filled out the multi‐dimensional emotional scale to measure baseline emotions before they put on headphones to watch the corresponding video. After watching the video, participants in all conditions completed measures of (a) current basic emotions, (b) core characteristics of awe, (c) small‐self, and (d) self‐other inclusion, and then clicked the link to log in to the game lobby to complete the online public goods dilemma game. There were 12 rounds in the game. After all the experimental tasks were completed, the investigator distributed gifts to the participants for their participation.

#### Experimental materials

##### Video material

The videos for the positive awe condition and the neutral emotion condition were the same as in Study 2. The video for the negative awe condition was a 4‐min video clip of threatening natural phenomena (e.g., tornados, lightning strikes, hurricanes, volcanoes) (Keltner & Haidt, 2003).

##### Current emotion states

These were the same as in Study 2.

##### Core characteristics of awe

This study adopted the three questions used in the study conducted by Stellar et al. (2017) to measure participants' perception of the two core characteristics of awe, namely perceived vastness and need for accommodation. The specific items were as follows: “*I feel vast and broad (physically or emotionally)*.” “*I feel that there is something beyond my current understanding*.” and “*I feel that I see the world from a different perspective*.”, were rated on a 7‐point Likert scale, ranging from 1 (totally disagree) to 7 (totally agree). The first item measured perceived vastness, while the average scores of the second and third items (*r* = .61, *p* < .001) were utilized to measure the need for accommodation.

##### Small‐self

The statement “*according to your feelings at the moment*” was added to the instruction of the small‐self questionnaire used in Study 1 to emphasize the participants' current experience. In this study, the α coefficient of the scale was .68.

##### Self‐other inclusion

The statement “*according to your feelings at the moment*” was added to the instruction of the self‐other inclusion questionnaire used in Study 1 to emphasize the participants' current experience.

##### Cooperative behavior

This was the same as in Study 2.

### Results and discussion

#### Manipulation checks

The mean and standard deviation of the score of basic emotions after induction and awe core characteristics of the three groups are shown in Table 3.

**TABLE 3:** Descriptive statistics of basic emotions after induction and awe core characteristics of the three groups (*M* ± *SD*).

Variables	Neutral group (*n* = 55)	Positive awe group (*n* = 49)	Negative awe group (*n* = 47)
Anger	1.59 ± 0.14	1.30 ± 0.15	1.65 ± 0.15
Sadness	1.46 ± 0.13	1.35 ± 0.14	1.78 ± 0.14
Awe	2.39 ± 0.24	3.13 ± 0.26	3.51 ± 0.27
Disgust	1.58 ± 0.15	1.30 ± 0.16	1.60 ± 0.16
Happiness	3.30 ± 0.25	3.28 ± 0.26	2.87 ± 0.27
Fear	1.51 ± 0.17	1.80 ± 0.19	2.17 ± 0.19
Vastness	3.95 ± 1.90	5.67 ± 1.59	5.51 ± 1.57
Accommodation	4.28 ± 1.66	4.98 ± 1.50	4.81 ± 1.47

While controlling for gender and age, a multivariate ANOVA was carried out on basic emotions before and after induction. The results showed that there was no significant difference in several basic emotions across the three groups at baseline. On post‐inducted emotional scores, the group main effect of awe [*F*(2,146) = 5.06, *p* = .008, *η* ^*2*^ = .07] and fear [*F*(2,146) = 3.23, *p* = .043, *η* ^*2*^ = .04] were significant. The awe emotion of the positive and negative awe groups was significantly higher than that of the neutral group (*M*_positive awe_−*M*_neutral_ = 0.73, *p* = .040; *M*_negative awe_−*M*_neutral_ = 1.12, *p* = .002), and the fear of the negative awe group was significantly higher than that of the neutral group (*M*_negative awe_−*M*_neutral_ = 0.66, *p* = .012), while the ratings of the other four emotions did not differ significantly between the three conditions. On the two core characteristics of awe, the group main effect of “vastness” [*F*(2,146) = 16.50, *p* < .001, *η* ^*2*^ = .184] and “accommodation” [*F*(2,146) = 3.47, *p* = .034, *η* ^*2*^ = .045] were both significant, in which the perceived vastness of positive awe and negative awe groups were significantly higher than the neutral group (*M*_positive awe_−*M*_neutral_ = 1.71, *p* < .001; *M*_negative awe_−*M*_neutral_ = 1.60, *p* < 0.001), the accommodation needs of the positive awe group were significantly higher than those of the neutral emotion group (*M*_positive awe_−*M*_neutral_ = 0.75, *p* = .014), and the accommodation needs of the negative awe group were marginally significantly higher than those of the neutral emotion group (*M*_negative awe_−*M*_neutral_ = 0.58, *p* = .061), indicating that the awe‐inducing operation was valid. The results of the sensitivity analysis (power = .80, *α* = .05) indicated that the effect size of the experimental group (*η* ^*2*^ = .140) was greater than the lowest detectable effect size for this sample size (*η* ^*2*^ = .066).

#### Influence of different valence of awe on cooperative behavior in the public goods dilemma

**FIGURE 3:**
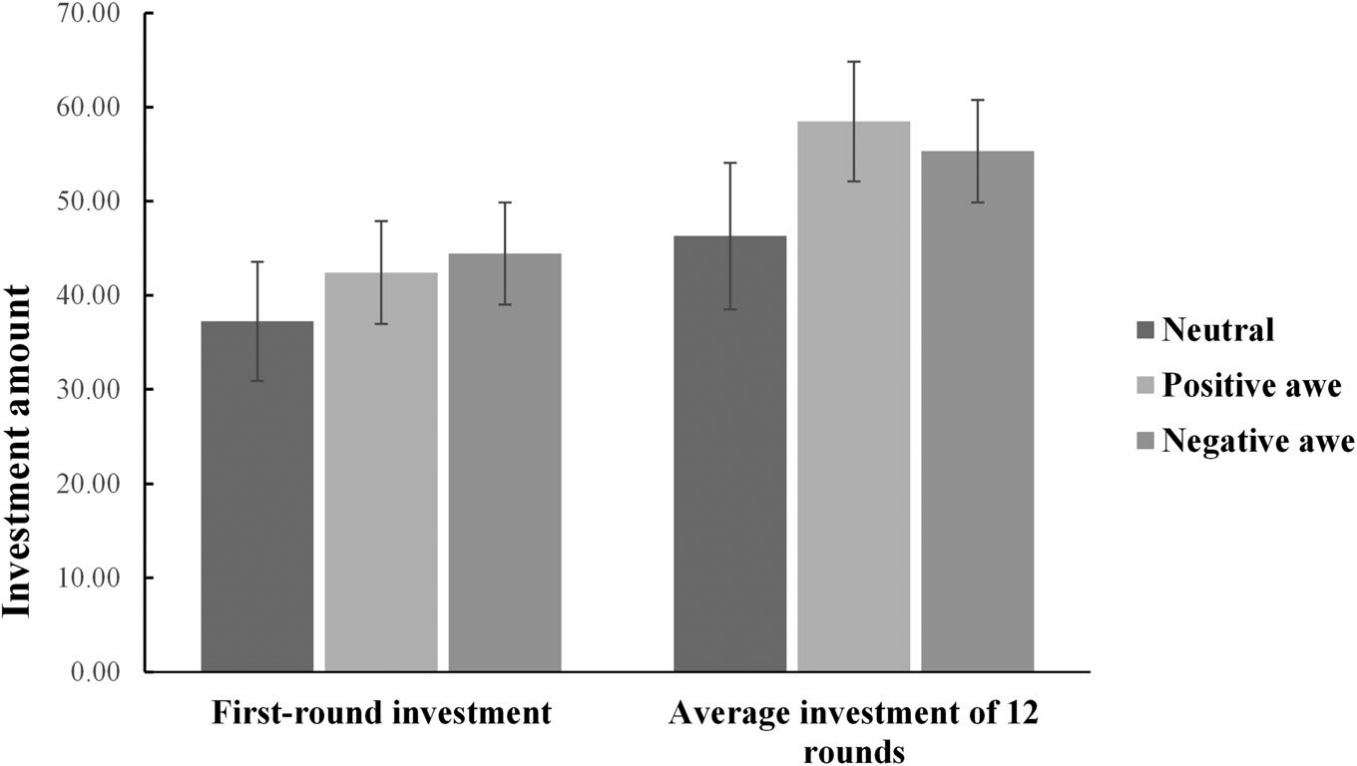
Average investment of 12 rounds and the first‐round investment of different emotional groups. The error bar in the figure represents the standard error.

Under the premise of controlling for gender and age, a multivariate ANOVA was conducted. The results showed that the group main effect on the average investment of 12 rounds was significant [*F*(2,146) = 4.19, *p* = .017, *η* ^2^ = .054]. The average amount of positive awe group (*M*_positive awe_−*M*_neutral_ = 12.02, *p* = .007) and negative awe group (*M*_negative awe_−*M*_neutral_ = 9.21, *p* = .041) were significantly higher than that of the neutral emotion group, and the main effect of the first round of investment was not significant [*F*(2,146) = 1.05, *p* = .351]. That is, both positive and negative awe emotions of different valence can significantly increase the average amount of multiple rounds of investment, but their effectiveness on cooperative behavior compared with the neutral emotion group is not reflected in the index of the first round of investment. The investment status of each group is shown in Figure 3. The results of the sensitivity analysis (power = .80, *α* = .05) indicated that the effect size of the experimental group (*η* ^2^ = .054) was greater than the lowest detectable effect size for this sample size (*η* ^2^ = .040).

#### The role of small‐self and self‐other inclusion in the effects of awe with different valence on cooperative behavior

We performed a multiple mediation analysis (Preacher & Hayes, 2008) to ascertain the specific indirect effects of small‐self and self‐other inclusion in the relationship between different valence types of awe and cooperative behaviors. Taking gender and age as control variables, the experimental group as the independent variable (neutral emotion group, positive awe group, and negative awe group), small‐self and self‐other inclusion as mediator variables, and the average investment of 12 rounds as the dependent variable, the mediating effect test of the multi‐category independent variables was carried out (the neutral emotion group as the reference level, Fang et al., 2017). We conducted this analysis using Model 4 of the PROCESS macro for SPSS (Hayes & Preacher, 2014) using 5000 bootstrap samples.

**FIGURE 4:**
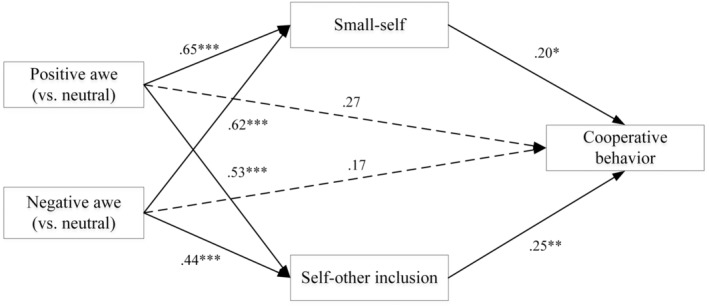
Parallel mediating effect of small‐self and self‐other inclusion between awe and cooperative behavior (positive awe and negative awe vs. neutral).

The results of the multiple mediation analysis showed that the positive awe (in contrast to the neutral emotion group) positively predicting small‐self (*β* = .65, *p* < .001) and self‐other inclusion (*β* = .53, *p* < .001), the negative awe (in contrast to the neutral emotion group) positively predicting small‐self (*β* = .62, *p* < .001) and self‐other inclusion (*β* = .44, *p* < .001), and small‐self (*β* = .20, *p* = .015) and self‐other inclusion (*β* = .25, *p* = .002) positively predicted cooperative behaviors. The 95% bias‐corrected confidence interval for the mediation for small‐self and self‐other inclusion in the relationship between the positive awe (in contrast to the neutral emotion group) and cooperative behaviors were [0.25, 6.58] and [0.52, 6.59], respectively, the relationship between the negative awe (in contrast to the neutral emotion group) and cooperative behaviors were [0.25, 5.90] and [0.17, 5.79], respectively, all excluding 0, suggesting that the small‐self and self‐other inclusion mediated the effect of the positive awe and the negative awe (in contrast to the neutral emotion group) on cooperative behaviors. The mediating effects of small‐self and self‐other inclusion accounted for 24.6% and 25% of the total effect of the positive awe (in contrast to the neutral emotion group) on cooperative behaviors, and accounted for 30.5% and 27.1% of the total effect of the negative awe (in contrast to the neutral emotion group) on cooperative behaviors, respectively. Figure 4 illustrates the mediation model and provides path coefficients.

We also set the negative emotion group as the reference level, to ascertain the specific effects of small‐self and self‐other inclusion in the relationship between the positive awe and cooperative behaviors. After controlling for gender and age, the results showed that the positive awe (in contrast to the negative awe group) on small‐self (*β* = .03, *p* = .883), self‐other inclusion (*β* = .09, *p* = .652) and cooperative behaviors (*β* = .10, *p* = .626) were nonsignificant. The 95% bias‐corrected confidence interval included zero, with [−0.08, 0.10] and [−0.07, 0.14] respectively, suggesting that there are no significant differences in the pathways through which positive and negative awe influence cooperative behaviors.

Study 3 further supported the parallel mediating role of small‐self and self‐other inclusion in the relationship between awe and cooperative behavior in the public goods dilemma by inducing the emotional state of awe in behavioral experiments. In addition, the negative awe group was added to the experimental group, in an attempt to explore whether there are differences in the effects of various awe valence types on cooperative behavior. The research results show that both positive and negative awe can affect cooperative behavior indirectly through the mediating effect of small‐self and self‐other inclusion, which further supports H2 and H3 from the perspective of emotional state.

## GENERAL DISCUSSION

Always be in awe makes behavior self‐disciplined. Awe is prosocial and self‐transcendent in nature and can restrict and regulate individual behavior. Given that awe can trigger relative diminishment of the individual self, blur boundaries, and enhance one's connection to others (Campos et al., 2013; Shiota et al., 2007; Stellar, 2021), we reasoned that awe could promote cooperation through small‐self and self‐other inclusion.

We conducted three studies to comprehensively explore the relationship between awe and cooperation and the role of small‐self and self‐other inclusion across different perspectives of emotional traits, states, valence, behavioral propensity, and decision‐making. The findings provide a series of supports for the role of awe in facilitating cooperation from different aspects. Namely, these findings demonstrated that individuals with higher dispositional tendencies to experience awe exhibited at higher propensities to cooperate (Study 1); positive and negative awe emotions of different valence can promote cooperative behavior in the dilemma of public goods (Study 2 and Study 3); and the existence of a parallel mediating role of small‐self and self‐other inclusion in the relationship between awe and cooperation (Study 1 and Study 3).

The present work's comprehensive exploration of the relationship between awe and cooperation from multiple dimensions contributes to the growing literature outlining the positive effect of emotion on social behavior. It extends the current knowledge on the self‐transcendent attributes of awe and furnishes empirical evidence for discussing the functions of self‐transcendent emotions. The exploration of the underlying mechanisms by which awe affects cooperation provides further validation of the pathways by which the social function of awe works, and supports the model of group‐based control. Moreover, the results of this study offer valuable insights into effectively promoting friendly cooperative interactions among adolescents through the cultivation of individual emotions at the practical level.

### Relationship between awe and cooperation from different perspectives

The study supported the facilitative effect of awe on cooperation from the trait and emotional state of awe, as well as the positive and negative valence of awe. These findings comprehensively supported H1 and H4, which was consistent with Perlin and Li (2020) who elaborated that awe enhances attention not only to other‐oriented concerns but rather to collective concerns, which balance the interests of the self and the other, and with the multiple findings that negative awe also promotes prosocial behavior (Piff et al., 2015; Seo et al., 2022; Wang et al., 2022), and further robustly confirms the positive social function of self‐transcendent awe.

Negative awe triggered by certain adverse conditions, such as disasters, may lead individuals to perceive each other as members of the same category and enhance feelings of social cohesion and mutual support (Levine & Manning, 2013; Wang et al., 2022). Therefore, similar to positive awe, emotional experience of negative awe enables individuals to experience a strong connection with others or a collective (Bai et al., 2017), urging us to transcend any one person's individual interest in pursuit of mutual interdependence. This further encourages active participation in collective action and facilitates cooperative interactions among individuals (Piff et al., 2015; Stellar et al., 2018). Furthermore, these research findings further support the model of group‐based control, suggesting that individuals may use social self, specifically engaging in collective actions, to buffer the feeling of loss of control and thereby regain a sense of control (Fritsche et al., 2013).

In Study 3, the awe‐inducing group significantly improved the average investment of 12 rounds in the public goods dilemma but did not increase the amount of the first‐round investment. On the one hand, this may be due to the supply point setting of 200 to the return structure of public goods, which makes the first‐round investment highly tentative, and most participants chose to invest just up to the amount of the supply point (around 50) in the first round. Alternatively, it may be that the awe emotion induced by the video is an individual's transient emotional state—its impact on behavior is time‐sensitive, and the impact effect weakens in the later stage. Therefore, in Study 2, participants' cooperative behavior was measured immediately after inducing awe and it was found that awe significantly increased in the first round of investment. Future research can continue to explore the role of awe in promoting cooperation with multiple indicators of cooperative behavior.

### Mediating effect of small‐self and self‐other inclusion

The questionnaire and behavioral experiment methods were combined to provide robust evidence for the mediating effect of small‐self and self‐other inclusion in the relationship between awe and cooperation, and these results support H2 and H3.

When faced with majestic mountains, contemplating the vast universe, or encountering extraordinary and ineffable things, there is an acute awareness of the vastness of the outside world and our own insignificance. At this time, the power of the self weakens so that people feel part of a larger collective or environment and devote themselves to collective activities (Shiota et al., 2007; Stellar, 2021). Simultaneously, the experience of awe blurs the boundaries between people and produces the power of integration with the outside world in the form of outward expansion (Rankin et al., 2020; Stellar, 2021). As a result, individuals can feel a stronger and broader affiliation, enhancing their overlapping relationship representation with others, and then pay more attention to collective or others' interests (Rankin et al., 2020). Thus, the hypothesis that awe can influence cooperation through the mediating effect of small‐self and self‐other inclusion is validated, further supporting the two sub‐components encompassed within self‐transcendence.

### Pathways of awe of different valence on cooperative behavior

Regarding H5, the results of Study 3 show that there is no difference in the influence of negative and positive awe on cooperative behavior and their influence pathways; both of them can indirectly affect the cooperative behavior of individuals through the mediating effect of small‐self and self‐other inclusion. This result validates H5 and aligns with findings from previous research regarding the self‐weakening effects of negative awe and its impact on collective group identity (Seo et al., 2022; Wang et al., 2022).

Although positive and negative awe are elicited by distinct stimuli, previous research has indicated that awe of different valence indeed presents variations in psychological experience evaluation (Chaudhury et al., 2021; Gordon et al., 2017). According to existing research results, the disparities in the effects of awe with different valence might be more prominent at the sense of meaning in life (Rivera et al., 2019) or one's attitude towards others' social norm violations (Lv et al., 2023).

Regarding the pathways through which awe of different valence influences cooperation, in the face of natural disasters, the identity of humans as a group is prominent and the community is easy to form, which enhances the inclusion of self and other identities. Additionally, there is no doubt that the feelings of negative awe or threatening stimulus can also elicit a diminished sense of self (Rivera et al., 2019; Vasey et al., 2012). Therefore, positive and negative awe will certainly affect the perception of the individual self and the relational self, and demonstrate stability in weakening self and strengthening connection in general, and then facilitate interpersonal cooperation through their mediating role. Further, negative awe is not a negatively univalenced awe experience but a mixed emotion that may have positive downstream effects (Chaudhury et al., 2021) and the results of this study contribute to the discourse on the positive effects of negative awe.

### Limitations and future directions

Despite comprehensively exploring the relationship between awe and cooperation using a variety of methods and from multiple perspectives, there are still some limitations that deserve attention.

Firstly, methodologically, this study only employed a cross‐sectional survey approach to examine the impact of awe traits on cooperative propensities. The cross‐sectional design limits the interpretation of causality and weakens the model's explanatory power. Secondly, during the conduct of the study, we did not perform prior sample size calculations based on the hypothetical model before data collection for Study 1; in Studies 2 and 3, the two behavioral experiments, we did not measure individual awe traits to control for their potential influence on the awe‐induction experiments, which has led to a certain degree of lack of rigor in hypothesis testing. Future research should consider incorporating measurements of individual awe traits to better elucidate the intricate roles of emotional states and emotional traits. Thirdly, the conclusions of Study 1 and Study 3 on the size of the mediating effect of small‐self and self‐other inclusion are not consistent, and the effect size of small‐self is relatively unstable. In recent years, some researchers have also proposed the authentic‐self hypothesis (Jiang & Sedikides, 2022) and quiet‐self hypothesis (Perlin & Li, 2020) of awe, which provide new perspectives for the influences of awe on the self. However, these theoretical disputes are in urgent need of scientific examination in future empirical research. Lastly, we conducted the study with high school students, which poses limitations to the representativeness of the adolescent population and the conclusions might have limitations when extrapolated to the whole population.

## CONCLUSION

In conclusion, this study validates the facilitating effect of awe on cooperation and the mediating role of small‐self and self‐other inclusion in their relationship from various aspects of emotional traits and states, emotionally positive and negative valence, behavioral propensities, and decision‐making, which offers robust evidence for asserting the positive functions of self‐transcendent emotional awe on social behavior.

## CONFLICT OF INTEREST STATEMENT

The authors declare no potential conflicts of interest.

## ETHICS STATEMENT

All study participants provided informed consent, and the study design was approved by the Ethics Review Board of the Social Sciences Office of Shanghai Normal University.

## Data Availability

The datasets for this study are available on request from the corresponding author.
